# Sinus pilonidalis in patients of German military hospitals: a review

**DOI:** 10.3205/iprs000061

**Published:** 2015-01-13

**Authors:** Janina Kueper, Theo Evers, Kai Wietelmann, Dietrich Doll, Jana Roffeis, Philipp Schwabe, Sven Märdian, Florian Wichlas, Björn-Dirk Krapohl

**Affiliations:** 1 Department of Plastic Surgery, Center for Musculoskeletal Surgery, Charité – Medical University of Berlin, Germany; 2 Military Hospital, Ulm, Germany; 3 Military Hospital, Berlin, Germany; 4 Department of Procto-Surgery, St. Marien Hospital, Vechta, Germany; 5 Center for Musculoskeletal Surgery, Charité – Medical University of Berlin, Germany

**Keywords:** pilonidal sinus, recurrence, family history, epilation, methylene blue, gentamycin, morphology

## Abstract

Pilonidal sinus disease (PSD) most commonly presents in young men when hair follicles enter through damaged epithelium and cause an inflammatory reaction. This results in the formation of fistular tracts. We reviewed studies based on a shared cohort of patients who presented at German military hospitals with PSD. The effect of the morphology of the sinus, perioperative protocol, and aftercare of the surgical treatment on the recurrence of PSD were evaluated. The drainage of acute abscesses before surgery, the application of methylene blue during surgery and open wound treatment were generally found to reduce the recurrence rate. A positive family history, postoperative epilation and primary suture as the healing method were found to elevate the recurrence rate. Long-term follow up of over 15 years was found to be a vital component of patient care as only 60% of the overall recurrences recorded had taken place by year 5 postoperatively.

## Zusammenfassung

Sinus pilonidalis findet sich am häufigsten bei jungen Männern, wenn Haarfollikel durch beschädigtes Epithel eindringen und eine entzündliche Reaktion der Weichteile verursachen. Dieses resultiert in der Bildung von fistelndem Gewebe. Wir überprüften Studien, die auf eine gemeinsame Gruppe von Patienten, die sich an deutschen Bundeswehrkrankenhäuser mit Sinus pilonidalis vorgestellt hatten, basiert sind. Die Wirkung der Morphologie des Sinus, des perioperativen Protokolls und der Nachsorge der chirurgischen Behandlung für das Wiederauftreten des Sinus wurden bewertet. Die Drainage von akuten Abszessen vor der Operation, die Anwendung von Methylenblau während der Operation und die offene Wundbehandlung reduzierten die Rückfallrate. Eine positive Familienanamnese, die postoperative Rasur und die Vernähung der Wunde als Heilmethode erhöhten die Rezidivrate. Die langfristige Verfolgung der Patienten über mehr als 15 Jahre stellte sich als ein wesentlicher Bestandteil der Patientenversorgung heraus, da nur 60% der Rezidive nach 5 Jahren verzeichnet worden waren.

## Introduction

First described by Herbert Mayo in 1833, pilonidal sinus disease (PSD) received major attention when found in large numbers of soldiers during World War II leading to its nick name – “Jeep Disease” [1], [2]. PSD has been described primarily in young men [3]. Both environmental and genetic components have been postulated to play a role in the etiology of the disease [4], [5], [6], [7]. Most commonly it appears that hair pierces through damaged epithelial layers of the opposing flap of skin in the presacral region, leading to an inflammatory reaction in the soft tissue above the Os sacrum. Fistular tissue accommodating the hair is formed, resulting in sinuses draining exudative fluids at the skin surface [8], [9]. With the epithelialization of the fistular tracts, a spontaneous remission becomes unlikely. The treatment of PSD therefore focuses primarily on the surgical removal of the affected tissue with the removal of all hair nests, fistular tracts and sinuses [10]. Many studies examining the development, presentation, timeline, treatment, and long-term follow up in PSD have been presented over the years. Few however examine all of these aspects in a homogenous cohort of patients at a long-term follow up after approximately 15 years. We aim to explore, interconnect and review the research performed by our workgroup over the years on a homogenous cohort of patients recruited from German military hospitals (Bundeswehrkrankenhäuser) and compare it to the literature. 

## Material and methods

All studies examined in this review received the Medical Ethics Committee’s approval. This retrospective review was exempt from our institution’s Medical Ethics Committee approval. 

All studies presented by our workgroup describing the identical group of patients surgically treated for PSD in one of three hospitals of the German armed forces, the Bundeswehr, were examined. The group represents a relatively homogenous cohort of patients due to the hospitals military backgrounds. 

The patients were originally selected through a query of patients treated with surgery for a pilonidal sinus between 1980 and 1996 at the Bundeswehr Institute for Military Medical Statistics and Epidemiology in Remagen. Patients were confirmed as eligible for enrollment in the study according to ICD codes, operative, referral and discharge notes. To not exclude patients with acute PSD who presented without visible sinuses, the histological specimens of these patients were reviewed for hair or fistulous tracts. Patients who showed histological evidence of PSD were subsequently added to the original patient cohort, leading to a total of 1960 patients eligible for study enrollment. For a variety of projects, differing sample sizes were calculated with the aim to achieve sufficient statistical power. Depending on the study, a varying number of patients was drawn at random to be contacted for a telephone interview. The focus of these interview differed, though the main focus throughout all studies remained recurrence. A recurrent case of PSD was defined to be confirmed either through the diagnosis of a doctor, a report of a surgical re-intervention by in- or excision, or the report of a formation of a new sinus and the presence of hair in a sinus opening. In addition, a patient was classified to have a recurrence if a minimum of two other criteria were fulfilled – erythema, edema, pain, or discharge around the presacral region.

The results of our studies were compared to the literature.

## Review of results

### Etiology

To determine the effect of a positive family history of PSD on the timeline, development and severity of the condition, 578 patients were interviewed on the phone. A family history of PSD in first- and second-degree relatives was determined, as well as the recurrence rate after a mean 15.4 years after surgical treatment. 12% of the patients reported relatives affected by PSD. This subgroup of patients was determined to have an earlier development of the disease and a recurrence rate of 52% at the time point of the telephone interview when compared to patients without a family history of PSD [11]. Only 3% of the patients who presented at one of the three hospitals gave a history of trauma, making traumatic disruptions of the skin’s integrity an unlikely cause of the condition [12].

### Presentation and intervention

An examination of the original patient cohort numbering 1,962 individuals revealed that patients presented with 1–16 sinuses prior to their surgical treatment. Patients diagnosed with a clinically chronic pilonidal sinus were shown to have an earlier onset of disease when compared to patients who presented with clinically acute pilonidal sinuses. Sinuses could be demonstrated to be formed primarily in a dermatologically vulnerable phase before the 22^nd^ year of life. Sinus number was shown to increase significantly with time in acute abscending PSD, but not in chronic PSD [13]. 

The interview of 498 patients interviewed after a mean 15.3 years demonstrated an increase in treatment volume of an average 5% per year, with open wound healing being an increasingly used method of healing after surgical treatment of PSD over primary suture [14].

Overall, nicotine abuse, patients with just one sinus and patients who had suffered from PSD for less than 5 months showed a higher rate of conversion to a clinically acute abscending pilonidal sinus than non-smokers who had suffered from PSD for more than 5 months [15]. Patients who fulfill criteria which have been correlated with a conversion from a sub-acute or chronic disease state to an acute disease state requiring emergent intervention should therefore preferably be recommended for prompt elective surgery.

### Aftercare

A total of 504 patients from the original patient cohort were interviewed on the implementation and effect of a postoperative aftercare protocol focusing on postoperative razor epilation of the hair surrounding the area of the surgically removed pilonidal sinus. The recurrence rate of PSD in patients who shaved for a mean 7.5 months postoperatively was 30.1% – significantly higher than the recurrence rate of 19.7% present in patients who did not shave [16]. Razor epilation may add additional dermatological microtrauma to the presacral area, increasing the risk of a renewed piercing of hair into the skin rather than decreasing it.

### Recurrence of PSD

Given the overall results of our cohort study, a 5–10 year follow up of patients who were treated for PSD appears to be the gold standard as only 15–20% of the recurrences will have occurred within the first postoperative year. 60% of the recurrences will have presented by year five, making it the barely adequate standard for a discussion of the main adverse outcome of the treatment of PSD [17]. 

A variety of risk factors for the recurrence of PSD was determined through the interview of selected subgroups of patients fulfilling the respective study criteria.

After the interview of 498 patients a mean 15.3 years after their surgical treatment, open wound healing could be demonstrated to have much better definite healing rates when compared to primary suture (16.8 vs. 31%). A BMI above 25 was not found to correlate with an increased rate of recurrence, whilst nicotine abuse was [14]. A second interview found no additional effect of nicotine abuse on wound dehiscence and delayed healing, but a potential protective effect of an increased BMI for the such after primary suture [18]. A greater deposit of fatty tissue in the presacral area and buttocks was hypothesized to allow for a closure with less tension on the wound edges. 

Given the classically described varied presentation of PSD as either acute, sub-acute or chronic, the patients with clinically inconspicuous PSD termed “asymptomatic sinuses” comprising 2.8% of the original patient cohort were further examined on an exploratory basis. Asymptomatic sinuses were found to be morphologically and histologically identical to sub-acute or chronic sinuses. The healing and recurrence rate did not differ from other forms of PSD. Given the lack of a necessity of treatment for the relief of pain and other symptoms, surgical treatment for the rare asymptomatic PSD should not be performed prophylactically [19].

59% of the patients in the original cohort presented with acute PSD, a difficult scenario given the infection and exacerbated inflammation of the sinus. Due to edema, some of the sinuses connecting to the fistular tract may swell shut and detain dye from identifying the entirety of the system. This may results in an incomplete removal of the pilonidal sinus during surgery. Incision and drainage prior to definitive surgery for acute PSD was postulated to decrease the inflammatory reaction of the tissue, allowing for better identification of the entire fistular tract system during the primary second part of the overall surgical treatment. After a mean 14.7 years after surgery, the recurrence rate in patients who had received an incision and drainage was 24% compared to a recurrence rate of 35% in patients who had not [20]. An incision and drainage procedure before the definitive surgical treatment of acute PSD was therefore shown to decrease the recurrence rate by 11%.

Acute abscess forming PSD as discussed above may at times require emergent surgical intervention for pain relief. In the interview of 546 patients, 16.8% of patients were found to have been treated on an emergent basis with same day surgery rather than elective surgery. This group of patients was found to have a significantly higher recurrence rate of 28.9% versus a recurrence rate of 17% in patients receiving elective surgery at 10 years follow up. This phenomenon was coupled with the observation of a decreased utilization of methylene blue during the surgical intervention, making it impossible to distinguish the primary risk factor for recurrence in acute PSD [21]. 

The utilization of methylene blue to dye the fistular tracts prior to their surgical removal was examined in an interview of 247 patients a mean 14.9 years after surgery. The application of the dye was found to reduce the recurrence rate by 14% from 30% in patients who did not receive methylene blue versus 16% in patients who did [22]. Whether this effect is caused by the antibacterial properties of the dye or its guiding visual component during surgery remains to be elucidated. 

The examination of topical gentamycin application after surgery to reduce the rate of recurrence possibly affected by postoperative complications such as infections was examined in a separate interview with 111 patients after a mean 15.4 years. The recurrence rate was found to differ only insignificantly [23]. Given the volatile microbiological environment of the presacral area, the effect of topical antibiotics on the rate of infection and wound healing difficulties should be examined further.

Overall, the median time until recurrence found in an interview with 205 patients after 14.8 years after their primary surgical intervention was found to be 1.8 years after open treatment and 2.7 years after primary suture. This time until recurrence was found to be decreased in second recurrence after a surgical intervention to treat the first recurrence [24]. 

## Discussion

The retrospective evaluation and telephone follow up performed 15 years after patients of German military hospitals received surgical treatment for PSD revealed interesting information on the development, morphology and postoperative outcome of the condition. 

No studies examining genetic factors in the development of PSD other than our exploratory project examining the influence of a family history on the development of the disease have been published to date. A more thorough examination of rare cases of PSD which develop at a very young age may further elucidate the hypotheses of congenital developments of the condition [25], [26]. Though embryologic malformations- and teratogenic factors have been discussed as potential causes of PSD, the condition is generally thought to be acquired through the inflammation of the tissue surrounding hair which has pierced the skin [4], [5], [6], [7], [8]. A history of Trauma, as discussed in select case reports, were not determined to correlate with PSD in our patient cohort [12], [27]. Risk factors for the development of PSD have been reported to be a plethora of hair in the gluteal cleft, a thick dermis, obesity, a deep gluteal cleft and a lack of hygiene [28], [29].

No studies examining the number or morphology of PSD in association with the timeline of the development of the condition have been presented. Larger epidemiological studies however have been able to approximate the incidence and prevalence of the disease. Overall, 0.7% of the population are estimated to suffer from PSD [30]. 

Though we examined exclusively surgical treatment for PSD and found open treatment to be increasingly used by surgeons as a method of closure rather than primary suture, other studies have focused on conservative measures such as phenol injection for certain forms of the condition [14]. The injection of this antiseptic and keratolytic agents first proposed in the 1960s has been advocated by some, with healing rates being reported between 59.8–95.1% [31], [32], [33], [34], [35]. The low number of patients in the case series and the limited time of follow up however merit critical discussion.

In terms of aftercare, patients in our cohort who shaved the hair in their gluteal cleft surrounding the now excised pilonidal sinus showed significantly higher recurrence rates than those who did not. Given the important role of hair in the etiology of the disease, contemporary studies have focused on the laser epilation of hair instead, a technology which was undeveloped at the time point our patients were treated. Laser hair removal of the natal cleft has proven to be a successful adjunct to surgical therapy for both primary and recurrent PSD, reducing the rate of recurrence and wound complications [36], [37], [38], [39].

Given the overall results of our cohort study, a 5–10 year follow up of patients who were treated for PSD appears to be the gold standard as only 15–20% of the recurrences will have occurred within the first postoperative year. 60% of the recurrences will have presented by year five, making it the barely adequate standard for a discussion of the main adverse outcome of the treatment of PSD [20]. 

A variety of risk factors for the recurrence of PSD were determined through the interview of select subgroups of patients fulfilling the respective study criteria. Risk factors for recurrence of PSD reported in the literature have been familiar disposition, obesity, female sex, number of sinuses, the cavity diameter and primary closure [40], [41], [42]. A weakness of our project were a lack of studies examining the risk factors of postoperative complications such as seromas, hematomas, wound-infections and dehiscences and their effect on long term recurrence rates. Select studies reported the use of a drain, D-flaps, distance of the lateral orifice from the midline of the cleft and a recurrent pilonidal sinus to be predictive of postoperative complications [42], [43], [44], [45].

## Conclusions

Though the longitudinal cohort project examining PSD in patients of German military hospitals was limited in its significance by its retrospective nature, the follow up extending over a 15 year period allowed for insight in long-term recurrence rates and contributing factors of adverse postoperative outcomes. Future prospective clinical studies should further elucidate the primary challenges found in the treatment of PSD: the avoidance of postoperative complications and recurrence. 

## Notes

### Competing interests

The authors declare that they have no competing interests.
